# Association between lncRNA *H19* rs217727 polymorphism and the risk of cancer: an updated meta-analysis

**DOI:** 10.1186/s12881-019-0904-x

**Published:** 2019-11-21

**Authors:** Xue Wang, Jialing Zhong, Fang Chen, Kang Hu, Suhong Sun, Yuanxiu Leng, Xumei Chen, Fengjiao Gan, Yana Pan, Qing Luo

**Affiliations:** 1 Department of Oncology, Affiliated Hospital of Zunyi Medical University, No.149 Dalian Road, Zunyi, 563003 China; 2 Clinical Laboratory, Sichuan Academy of Medical Science and Sichuan Provincial People’s Hospital, School of Medicine, University of Electronic Science and Technology of China, Chengdu, 610072 China; 3 Breast & Thyroid Disease Medical Center, Affiliated Hospital of Zunyi Medical University, Zunyi, China; 4 The people’s Hospital of Tongnan District, Chongqing, China

**Keywords:** Cancer risk, *H19*, rs217727, Polymorphism, Meta-analysis

## Abstract

**Background:**

We have performed this study to evaluate the association between *H19* rs217727 polymorphism and the risk of cancer.

**Methods:**

An odds ratio (OR) with a 95% confidence interval (CI) was applied to determine a potential association.

**Results:**

A total of 17 case–control publications were selected. This meta-analysis showed that *H19* rs217727 has a significant increased association with cancer risk in allelic, homozygous, heterozygote, dominant and recessive models (T vs C: OR = 1.16, 95% CI = 1.06–1.27, *I*^*2*^ = 75.7; TT vs CC: OR = 1.29, 95% CI = 1.06–1.56, *I*^*2*^ = 71.6; CT vs CC: OR = 1.15, 95% CI = 1.01–1.31, *I*^*2*^ = 75.4; CT + TT vs CC: OR = 1.20, 95% CI = 1.05–1.36, *I*^*2*^ = 76.5; TT vs CT + CC: OR = 1.22, 95% CI = 1.02–1.45, *I*^*2*^ = 70.6;). In the subgroup analysis of smoking status, both smokers and nonsmokers showed an increase in cancer risk in allelic, homozygous, dominant and heterozygote models.

**Conclusion:**

This meta-analysis revealed *H19* rs217727 may influence cancer susceptibility.

## Background

Cancer has become a major public health problem and gives the second leading cause of death after cardiovascular and cerebrovascular disease. Therefore, identification of modifiable risk factors to slow cancer progression is crucial. Environmental factors, smoking [1], alcohol consumption [2], human papillomavirus (HPV) [3], and the Epstein-Barr virus (EBV) [4] was known to play a key role in the pathogenesis and tumorigenesis. In addition, single nucleotide polymorphisms (SNPs) were recognized to be associated with cancer development too. For example, CpG rs1190983, rs155247, and rs62382272 play an important role in oncogenesis in breast cancer [5], and the rs874945 in HOX transcript antisense RNA (*HOTAIR)* gene increases the risk of bladder cancer in Chinese population [6].

*H19* (Gene ID: 283120) is an imprinted gene, located on chromosome 11p15.5, close to the insulin-like growth factor 2 (*IGF2*) gene, which has 6 exons and can produce long non-coding RNA (lncRNA) with a length of 2326 bp. *H19* is mainly involved in the development of the embryo, showing high expression in the fetus, rapidly down-regulated after birth, and only continuously expressed in the heart and skeletal muscle in adults. However, *H19* was found to be highly expressed in a variety of cancers. Previous studies have demonstrated that increased levels of *H19* contributes to melanoma development and progression [7]. In addition, the introduction of the genome-wide association studies (GWAS) allowed for identification of an increased number of *H19* SNPs that were associated with various types of cancer. For instance, *H19* rs217727 has been reported to significantly increase the risk of gastric cancer [8], and colorectal cancer [9]. In addition, a large number of studies have found that *H19* lncRNA tag SNPs (rs217727, rs2839698, rs3741216, rs3741219, rs2107425, rs3024270, rs2735971, rs2071095) are related to the susceptibility of cervical cancer [10], breast cancer [11–15], bladder cancer [16–18], gastric cancer [8], lung cancer [19, 20], osteosarcoma [21], pancreatic cancer [22], and oral squamous cell carcinoma [23, 24]. Among them, rs217727 is located in the exon 5 of the *H19* gene. Some original studies and previous meta-analyses reported the relationship between *H19* rs217727 and cancer risk, but the results were inconsistent. In addition, several recently published studies provide the basis for updating data sets and more accurately evaluating the relationship between *H19* rs 217,727 and cancer risk. Thus, we performed meta-analysis to explore the association between *H19* polymorphisms and the risk of cancer.

## Methods

For this meta-analysis study, patient consent and ethical approval was not required. We performed this meta-analysis as per the Preferred Reporting Items for Systematic Reviews and Meta-Analyses (PRISMA) statement [25]. Two independent investigators participated in study selection and data extraction, and any disagreement was solved by discussion and reinterpretation of the data involved.

### Selection and exclusion criteria

The eligibility criteria were as follows: (1) case-control studies, in which the relation between *H19* rs217727 polymorphism and the risk of cancer was evaluated; (2) 2 or more studies focused on *H19* rs217727 polymorphism; (3) the genotype frequency was reported; (4) published as a full-text manuscript in the English language. We excluded meta-analysis, reviews, as well as the articles lack of healthy controls, or polymorphism type not detected.

### Literature and research strategy

We searched the databases Embase, PubMed, and Web of Science up to January 06, 2019 using the keywords “*H19* OR long noncoding RNA *H19*” AND “cancer OR tumor OR neoplasm” AND “mutation OR variant OR polymorphism”. Studies related to the association of *H19* rs217727 polymorphism and cancer risk were obtained. In addition, references and meta-analyses of the studies included were searched manually. The search strategy in PubMed are shown in Additional file 1.

### Data extraction and synthesis

Data was extracted and listed on the predesigned data extraction sheet included first author, publication year, country, ethnicity (Asian or Caucasian), source of control, type of cancer, type of polymorphism, number and genotyping distribution of cases and controls, genotyping method, smoking status and *P*-value of Hardy-Weinberg Equilibrium (HWE) in controls [26]. Authors involved were contacted and asked for data usage, when necessary.

### Quality assessment

The quality of the included studies was evaluated by two independent investigators according to the Newcastle Ottawa Scale (NOS) [27]. The points were awarded on selection (case definition adequate, representativeness of the cases, selection of controls, definitions of controls), comparability (comparability of cases and controls on the basis of the design or analysis) and exposure (ascertainment of exposure, uniform method of ascertainment, nonresponse rate) and the total score ranged from 0 to 9. Study with a score of more than 5 was included in the meta-analysis.

### Data analysis

We used the OR and 95% CI to present the strength of the association using an allelic model (T vs. C), homozygote model (TT vs. CC), heterozygote model (CT vs. CC), dominant model [(CT + TT) vs. CC] and recessive model [TT vs. (CT + CC)]. Meta-analysis was conducted if 2 or more studies were performed for the same type of polymorphism. Initially, heterogeneity was evaluated by the Chi square-based Q-test, and *I*^*2*^ statistics. A value of *P* ≥ 0.1 and *I*^*2*^ ≤ 50% indicated that heterogeneity was absent, and the fixed-effect model was used. In other occasions, the random-effect model was used. Moreover, subgroup analyses were conducted based on ethnicity, type of cancer, source of controls, sample size, genotyping approach and smoking status. Evaluation of any publication bias was performed by Begg’s and Egger’s tests, when *P* < 0.1, publication bias was considered to exist. Sensitive analysis was performed by elimination of each study to observe the effect of a single study on the pooled OR. Statistical analysis was performed using Stata software version 12.0 (Stata Corporation, College Station, TX, USA).

## Results

### Study identification

**Fig. 1:**
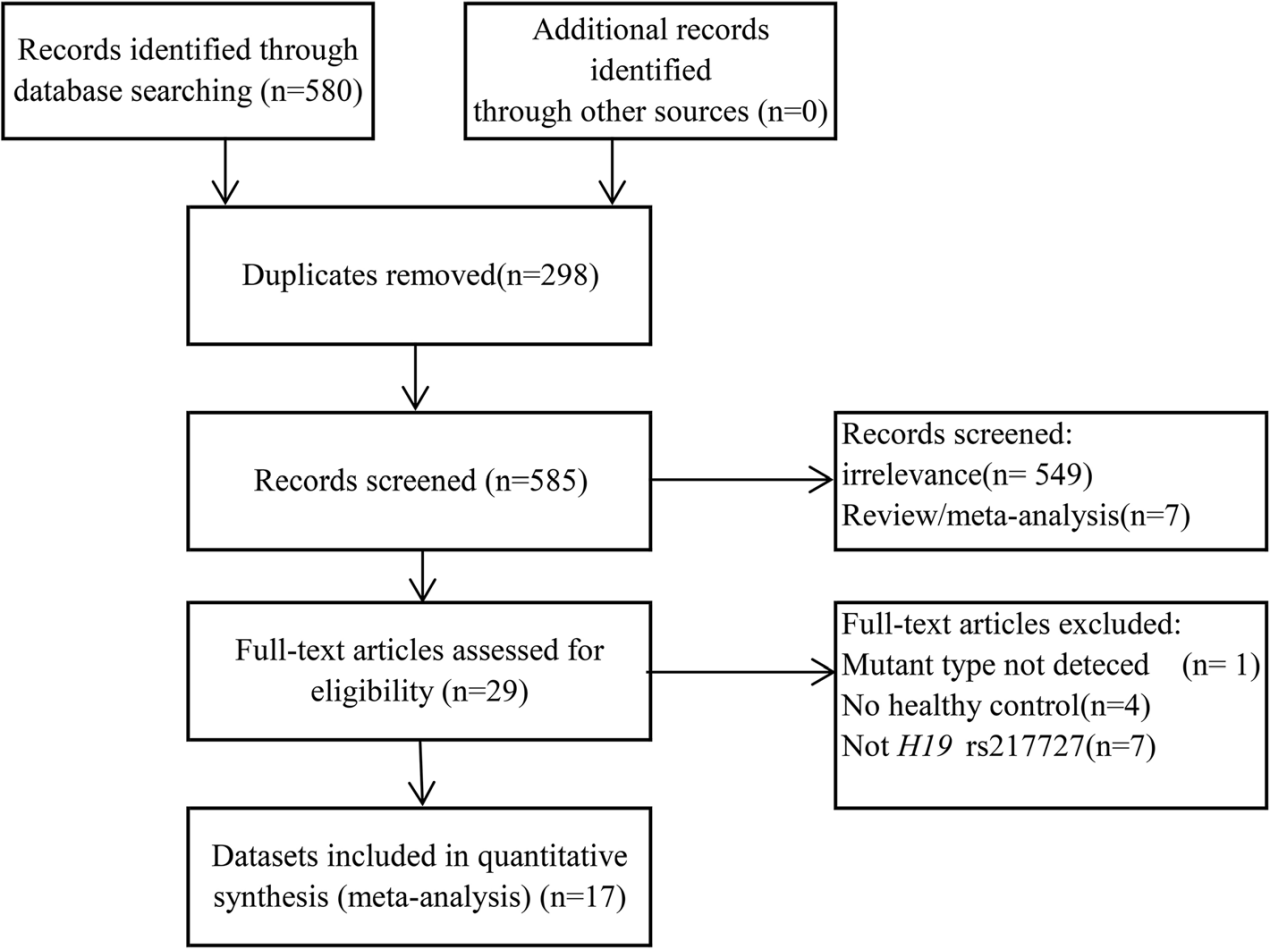
Study selection flowchart

In this meta-analysis, a total of 17 case–control publications [8–14, 16–19, 21–24], including 9166 cancer patients and 10,823 healthy controls were selected. A summary of data retrieval and selection is summarized in Fig. 1.

### Characteristics and quality of the study

In these 17 studies, 8 types of cancer were studied, including gastric cancer, breast cancer, lung cancer, bladder cancer, osteosarcoma, cervical cancer, oral squamous cancer, and digestive system tumors. Eight of the studies focused on general population and 9 on hospital data. All studies were performed in Asians, except one in Caucasians. The summary characteristics are described in Table 1. In addition, the relationship between smoking status and genetic polymorphism has been reported in only 4 studies [8, 17, 23, 24], and the summary characteristics are described in Table 2.

**Table 1:** Characteristics of included studies in the meta-analysis (rs217727 C>T)

Author	Year	Country	Ethnicity	Sample size (case/control)	Source of control	Cancer site and type	Genotype distribution						Genotyping method	*P* for HWE
							Case			Control				
							CC	CT	TT	CC	CT	TT		
Jin [10]	2016	China	Asian	246/284	PB	cervical cancer	117	103	26	169	99	16	MassArray	0.74
Li [9]	2016	China	Asian	1147/1203	PB	colorectal cancer	480	514	153	456	570	177	TaqMan	0.959
Xia [11]	2016	China	Asian	464/467	PB	breast cancer	160	156	148	139	212	116	CRS-RFLP	0.052
Hua [17]	2016	China	Asian	1046/1394	HB	bladder cancer	431	467	148	573	665	156	TaqMan	0.074
Yang [8]	2015	China	Asian	500/500	HB	gastric cancer	160	252	88	193	244	63	TaqMan	0.296
Verhaegh [16]	2008	Netherlands	Caucasian	177/204	PB	bladder cancer	114	59	4	115	80	9	PCR-RFLP	0.288
Hu [22]	2017	China	Asian	416/416	HB	pancreatic cancer	133	200	83	128	196	92	TaqMan	0.302
Guo [23]	2017	China	Asian	362/740	PB	oral squamous cell carcinoma	101	181	80	255	348	137	BeadChip	0.342
Lin [12]	2017	China	Asian	1005/1020	HB	breast cancer	403	471	131	465	450	105	SNPscan	0.801
He [21]	2017	China	Asian	193/383	HB	osteosarcoma	79	102	12	195	165	23	TaqMan	0.121
Hassanzarei [13]	2017	Iranian	Asian	230/240	PB	breast cancer	71	132	27	125	113	2	PCR-RFLP	0
Li [18]	2018	China	Asian	200/200	HB	bladder cancer	51	140	9	84	90	26	TaqMan	0.806
Yuan [24]	2018	China	Asian	431/984	PB	oral squamous cell carcinoma	186	194	51	488	423	73	MassArray	0.151
Cui [14]	2018	China	Asian	1488/1675	PB	breast cancer	611	692	185	685	773	217	TaqMan	0.963
Li [19]	2018	China	Asian	555/618	HB	lung cancer	210	250	95	246	305	67	TaqMan	0.053
Abdollahzadeh [15]	2018	Iranian	Asian	150/100	HB	breast cancer	116	29	5	86	14	0	PCR-RFLP	0.452
Yin [20]	2018	China	Asian	556/395	HB	lung cancer	204	264	88	165	172	58	TaqMan	0.232

**Table 2:** Smoking status: characteristics of studies included in the meta-analysis

Author	Year	Cancer site and type	Smokers						Nonsmokers					
			Case			Control			Case			Control		
			CC	CT	TT	CC	CT	TT	CC	CT	TT	CC	CT	TT
Hua [17]	2016	bladder cancer	187	308	73	250	229	52	219	257	75	368	391	104
Yang [8]	2015	gastric cancer	44	60	20	49	68	24	116	186	74	144	167	48
Guo [23]	2017	oral squamous cell carcinoma	35	75	30	81	131	49	66	106	50	174	217	88
Yuan [24]	2018	oral squamous cell carcinoma	79	76	18	179	138	26	107	118	33	309	285	47
Yin [20]	2018	lung cancer	0	0	0	0	0	0	204	264	88	165	172	58

### Quality assessment

**Fig. 2:**
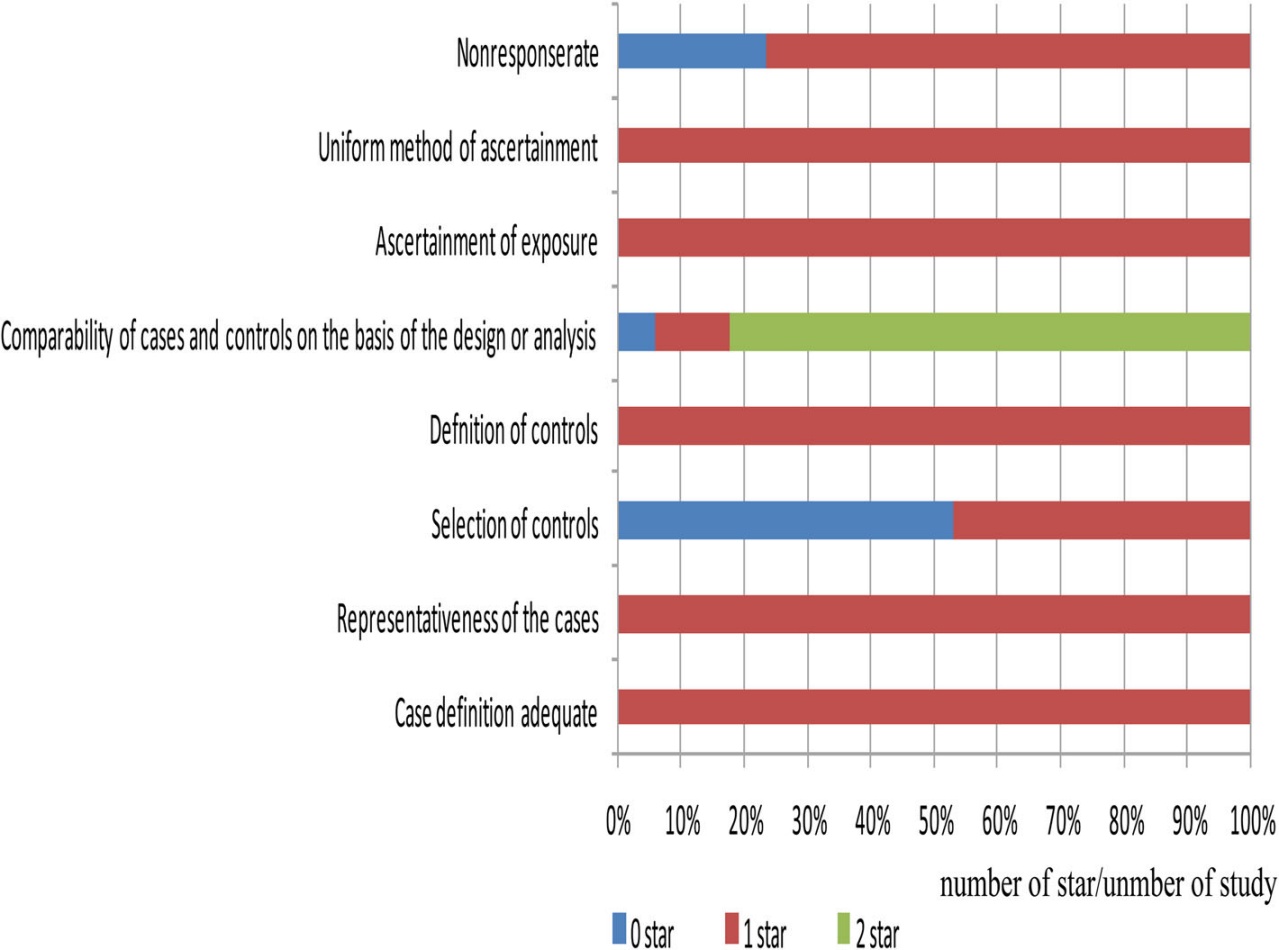
Graph of quality assessments

According to the NOS, detailed quality assessment for each study included are presented in Table 3, the score of each included study is more than 7 points, higher scores were associated with lower risks of bias. The percentage of quality assessment is presented in Fig. 2.

**Table 3:** Quality score assessment

Studies	Selection				Comparability	Exposure			Total
	Case definition adequate	Representativeness of the cases	Selection of controls	Definition of controls	Comparability of cases and controls on the basis of the design or analysis	Ascertainment of exposure	Uniform method of ascertainment	Nonresponserate	
Jin [10]	*	*	*	*	**	*	*	*	9
Li [9]	*	*	*	*	**	*	*	*	9
Xia [11]	*	*	*	*	**	*	*	*	9
Hua [17]	*	*	0	*	**	*	*	0	7
Yang [8]	*	*	0	*	**	*	*	*	8
Verhaegh [16]	*	*	*	*	**	*	*	*	9
Hu [22]	*	*	0	*	**	*	*	*	8
Guo [23]	*	*	*	*	**	*	*	0	8
Lin [12]	*	*	0	*	*	*	*	*	7
He2 [1]	*	*	0	*	**	*	*	*	8
Hassanzarei [13]	*	*	*	*	0	*	*	*	7
Li [18]	*	*	0	*	**	*	*	*	8
Yuan [24]	*	*	*	*	**	*	*	0	8
Cui [14]	*	*	*	*	*	*	*	0	7
Li [19]	*	*	0	*	**	*	*	*	8
Abdollahzadeh [15]	*	*	0	*	**	*	*	*	8
Yin [20]	*	*	0	*	**	*	*	*	8

*indicates a score of 1, **indicates a score of 2. The total score ranged from 0 to 9

### Statistical analysis

**Fig. 3:**
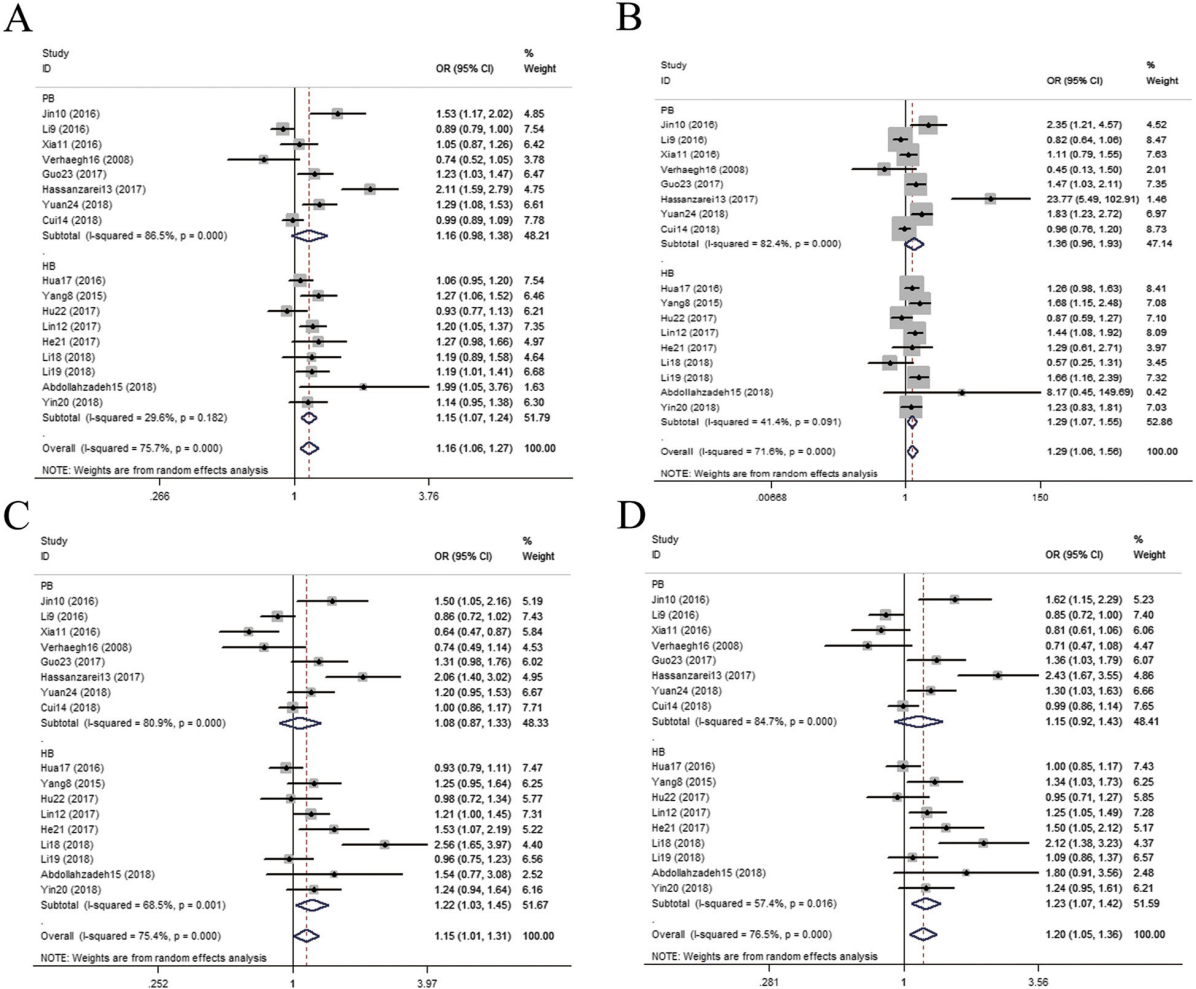
Forest plots for H19 rs217727 polymorphism associated with risk of cancer in subgroup analysis under hospital-based controls. **a** Allelic model (T vs. C), **b** homozygote model (TT vs. CC). **c** Heterozygote model (CT vs. CC). **d** Dominant model [(CT + TT) vs. CC]

As shown in Table 4, *H19* rs217727 was found to increase cancer risk in overall analysis under T vs C (OR = 1.16, 95% CI = 1.06–1.27, *I*^*2*^ = 75.7), TT vs CC (OR = 1.29, 95% CI = 1.06–1.56, *I*^*2*^ = 71.6), CT vs CC (OR = 1.15, 95% CI = 1.01–1.31, *I*^*2*^ = 75.4), CT + TT vs CC (OR = 1.20, 95% CI = 1.05–1.36, *I*^*2*^ = 76.5), TT vs CT + CC (OR = 1.22, 95% CI = 1.02–1.45, *I*^*2*^ = 70.6). When stratifying data by ethnicity, genotyping approach and type of cancer, the allelic, homozygote, heterozygote, dominant and recessive models of rs217727 were observed to increase cancer risk based on Asians (T vs C: OR = 1.18, 95% CI = 1.08–1.29, *I*^*2*^ = 75.3, TT vs CC: OR = 1.32, 95% CI = 1.09–1.59, *I*^*2*^ = 72.1, CT vs CC: OR = 1.18, 95% CI = 1.03–1.34, *I*^*2*^ = 75.9, CT + TT vs CC: OR = 1.23, 95% CI = 1.08–1.39, *I*^*2*^ = 76.4, TT vs CT + CC: OR = 1.24, 95% CI = 1.04–1.47, *I*^*2*^ = 71.4), subgroups for genotyping based on MassArray (T vs C: OR = 1.36, 95% CI = 1.16–1.60, *I*^*2*^ = 13.8, TT vs CC: OR = 1.96, 95% CI = 1.39–2.75, *I*^*2*^ = 0, CT vs CC: OR = 1.29, 95% CI = 1.05–1.57, *I*^*2*^ = 0.4, CT + TT vs CC: OR = 1.39, 95% CI = 1.14–1.71, *I*^*2*^ = 10.9, TT vs CT + CC: OR = 1.75, 95% CI = 1.26–2.42, *I*^*2*^ = 0) and oral squamous cell carcinoma (T vs C: OR = 1.26, 95% CI = 1.11–1.42, *I*^*2*^ = 0, TT vs CC: OR = 1.63, 95% CI = 1.25–2.12, *I*^*2*^ = 0, CT vs CC: OR = 1.25, 95% CI = 1.04–1.50, *I*^*2*^ = 0, CT + TT vs CC: OR = 1.32, 95% CI = 1.11–1.57, *I*^*2*^ = 0, TT vs CT + CC: OR = 1.42, 95% CI = 1.07–1.88, *I*^*2*^ = 28.1). *H19* rs217727 significantly increased the risk of lung cancer in the allelic, homozygote models (T vs C: OR = 1.17, 95% CI = 1.03–1.33, *I*^*2*^ = 0, TT vs CC: OR = 1.44, 95% CI = 1.07–1.94, *I*^*2*^ = 19.4), as well as breast cancer in the allelic model (T vs C: OR = 1.29, 95% CI = 1.02–1.62, *I*^*2*^ = 86.8). We also conducted subgroup analysis by source of controls and sample size, the pooled results showed that the allelic, homozygote, heterozygote and dominant model of rs217727 have a positive association with cancer risk in hospital-based controls, as shown in Fig. 3 (T vs C: OR = 1.15, 95% CI = 1.07–1.24, *I*^*2*^ = 29.6, TT vs CC: OR = 1.29, 95% CI = 1.07–1.55, *I*^*2*^ = 41.4, CT vs CC: OR = 1.21, 95% CI = 1.03–1.45, *I*^*2*^ = 68.5, CT + TT vs CC: OR = 1.23, 95% CI = 1.07–1.42, *I*^*2*^ = 57.4); Similarly, a positive relation was observed between the allelic, homozygous, dominant and recessive models and the risk of cancer when the case sample size ≥500 (T vs C: OR = 1.13, 95% CI = 1.04–1.22, *I*^*2*^ = 67.1, TT vs CC: OR = 1.27, 95% CI = 1.08–1.49, *I*^*2*^ = 63.6, CT + TT vs CC: OR = 1.13, 95% CI = 1.01–1.25, *I*^*2*^ = 66.4, TT vs CT + CC: OR = 1.25, 95% CI = 1.08–1.41, *I*^*2*^ = 56.4). As shown in Table 5, when stratifying data by smoking status, all the genetic models of rs217727 have a positive association with cancer risk in smokers, as well as in nonsmokers except in recessive model.

**Table 4:** Overall and subgroups meta-analysis of *H19* rs217727 (C > T) polymorphism and cancer risk

Overall and subgroups	NO.	T versus C			TT versus CC			CT versus CC			CT + TT versus CC			TT versus CT + CC		
		OR (95% CI)	P_Q_	I^2^(%)	OR (95% CI)	P_Q_	I^2^(%)	OR (95% CI)	P_Q_	I^2^(%)	OR (95% CI)	P_Q_	I^2^(%)	OR (95% CI)	P_Q_	I^2^(%)
Total	17	1.16 (1.06, 1.27)	0	75.7	1.29 (1.06, 1.56)	0	71.6	1.15 (1.01, 1.31)	0	75.4	1.20 (1.05, 1.36)	0	76.5	1.22 (1.02, 1.45)	0	70.6
Ethnicity																
Asians	16	1.18 (1.08, 1.29)	0	75.3	1.32 (1.09, 1.59)	0	72.1	1.18 (1.03, 1.34)	0	75.9	1.23 (1.08, 1.39)	0	76.4	1.24 (1.04, 1.47)	0	71.4
Caucasians	1	0.74 (0.52, 1.05)	NA	NA	0.45 (0.13, 1.50)	NA	NA	O.74 (0.49, 1.14)	NA	NA	0.71 (0.47, 1.08)	NA	NA	0.50 (0.15, 1.66)	NA	NA
Method																
TaqMan	9	1.07 (0.98, 1.17)	0.01	60.6	1.12 (0.92, 1.36)	0.01	63.2	1.12 (0.96, 1.31)	0	74.1	1.12 (0.98, 1.29)	0	72.6	1.06 (0.87, 1.31)	0	70.8
MassArray	2	1.36 (1.16, 1.60)	0.28	13.8	1.96 (1.39, 2.75)	0.53	0	1.29 (1.05, 1.57)	0.32	0.4	1.39 (1.14, 1.71)	0.29	10.9	1.75 (1.26, 2.42)	0.66	0
PCR-RFLP	3	1.44 (0.68, 3.05)	0	90.9	4.14 (0.21, 80.14)	0	89	1.32 (0.66, 2.64)	0	83.7	1.45 (0.63, 3.35)	0	89.4	3.60 (0.26, 49.72)	0	86.2
Others	3	1.17 (1.07, 1.28)	0.4	0	1.33 (1.11, 1.61)	0.42	0	1.01 (0.68, 1.51)	0	86	1.12 (0.84, 1.49)	0.01	77	1.33 (1.12, 1.57)	0.83	0
Cancer type																
Breast cancer	5	1.29 (1.02, 1.62)	0	86.8	1.56 (0.95, 2.56)	0	83	1.15 (0.84, 1.55)	0	84.2	1.27 (0.94, 1.71)	0	85.7	1.48 (0.98, 2.26)	0	80.3
Bladder cancer	3	1.01 (0.82, 1.25)	0.1	56.8	0.80 (0.40, 1.61)	0.06	64	1.20 (0.64, 2.23)	0	90.1	1.13 (0.68, 1.88)	0	85.9	0.63 (0.22, 1.80)	0	85.1
Digestive system cancer^a^	3	1.02 (0.82, 1.26)	0.04	81.6	1.05 (0.68, 1.62)	0.01	79.8	1.00 (0.79, 1.26)	0.08	61	1.01 (0.77, 1.34)	0.02	76.1	1.03 (0.76, 1.41)	0.04	68.5
Osteosarcoma	1	1.27 (0.98, 1.66)	NA	NA	1.29 (0.61, 2.71)	NA	NA	1.53 (1.07, 2.19)	NA	NA	1.50 (1.05, 2.12)	NA	NA	1.04 (0.50, 2.13)	NA	NA
Cervical cancer	1	1.53 (1.17, 2.02)	NA	NA	2.35 (1.21, 4.57)	NA	NA	1.50 (1.05, 2.16)	NA	NA	1.62 (1.15, 2.29)	NA	NA	1.98 (1.04, 3.78)	NA	NA
Oral squamous cell carcinoma	2	1.26 (1.11, 1.42)	0.72	0	1.63 (1.25, 2.12)	0.42	0	1.25 (1.04, 1.50)	0.65	0	1.32 (1.11, 1.57)	0.8	0	1.42 (1.07, 1.88)	0.24	28.1
Lung cancer	2	1.17 (1.03, 1.33)	0.73	0	1.44 (1.07, 1.94)	0.27	19.4	1.08 (0.84, 1.39)	0.18	44.4	1.15 (0.97, 1.37)	0.47	0	1.37 (0.89, 2.11)	0.08	67.6
Source ofcontrols																
Population-based	8	1.16 (0.98, 1.38)	0	86.5	1.36 (0.96, 1.93)	0	82.4	1.08 (0.87, 1.33)	0	80.9	1.15 (0.92, 1.43)	0	84.7	1.30 (0.98, 1.73)	0	77.4
Hospital-based	9	1.15 (1.07, 1.24)	0.18	29.6	1.29 (1.07, 1.55)	0.09	41.4	1.21 (1.03, 1.45)	0	68.5	1.23 (1.07, 1.42)	0.02	57.4	1.16 (0.93, 1.46)	0	64.7
Case sample size																
≥ 500	13	1.13 (1.04, 1.22)	0	67.1	1.27 (1.08, 1.49)	0	63.6	1.08 (0.96, 1.20)	0	65.2	1.13 (1.01, 1.25)	0	66.4	1.25 (1.08, 1.41)	0.01	56.4
< 500	4	1.36 (0.83, 2.23)	0	87.1	2.29 (0.31, 16.97)	0	88.2	1.57 (0.88, 2.80)	0	83.9	1.60 (0.87, 2.92)	0	85.8	1.77 (0.23, 13.44)	0	89

^a^Including colorectal cancer, gastric cancer and pancreatic cancer

**Table 5:** Smoking status: Meta-analysis of the association between the *H19* rs217727 polymorphism and cancer risk

Smoking status	NO.	T versus C			TT versus CC			CT versus CC			CT + TT versus CC			TT versus CT + CC		
		OR (95% CI)	P_Q_	I^2^(%)	OR (95% CI)	P_Q_	I^2^(%)	OR (95% CI)	P_Q_	I^2^(%)	OR (95% CI)	P_Q_	I^2^(%)	OR (95% CI)	P_Q_	I^2^(%)
smokers	4	1.29 (1.14, 1.46)	0.19	37.9	1.55 (1.17, 2.03)	0.41	0	1.48 (1.23, 1.77)	0.14	44.8	1.49 (1.26, 1.78)	0.11	49.8	1.25 (0.97, 1.61)	0.77	0
nonsmokers	5	1.21 (1.11, 1.32)	0.41	0	1.46 (1.22, 1.76)	0.28	21.1	1.21 (1.07, 1.38)	0.84	0	1.27 (1.12, 1.43)	0.64	0	1.31 (1.10, 1.55)	0.33	13.4

### Heterogeneity analysis

In this meta-analysis, heterogeneity was observed, we next performed the stratified analysis to evaluate the source of the heterogeneity. The heterogeneity decreased significantly or disappeared in genotyping approach of MassArray (T vs C:*P* = 0.28, *I*^*2*^ = 13.8, TT vs CC: *P* = 0.53, *I*^*2*^ = 0, CT vs CC: *P* = 0.32, *I*^*2*^ = 0.4, CT + TT vs CC: *P* = 0.29, *I*^*2*^ = 10.9, TT vs CT + CC: *P* = 0.66, *I*^*2*^ = 0), oral squamous cell carcinoma (T vs C:*P* = 0.72, *I*^*2*^ = 0, TT vs CC: *P* = 0.42, *I*^*2*^ = 0, CT vs CC: *P* = 0.65, *I*^*2*^ = 0, CT + TT vs CC: *P* = 0.8, *I*^*2*^ = 0, TT vs CT + CC: *P* = 0.24, *I*^*2*^ = 28.1) and lung cancer (T vs C:*P* = 0.73, *I*^*2*^ = 0, TT vs CC: *P* = 0.27, *I*^*2*^ = 19.4, CT vs CC: *P* = 0.18, *I*^*2*^ = 44.4). Furthermore, analyses of control subjects demonstrated that heterogeneity was significantly reduced in hospital-based controls in allelic models (T vs C: *P* = 0.18, *I*^*2*^ = 29.6). Nevertheless, heterogeneity was still present in other subgroups. In Table 4, an overview of all analyses is presented.

### Sensitivity analysis and publication bias

**Fig. 4:**
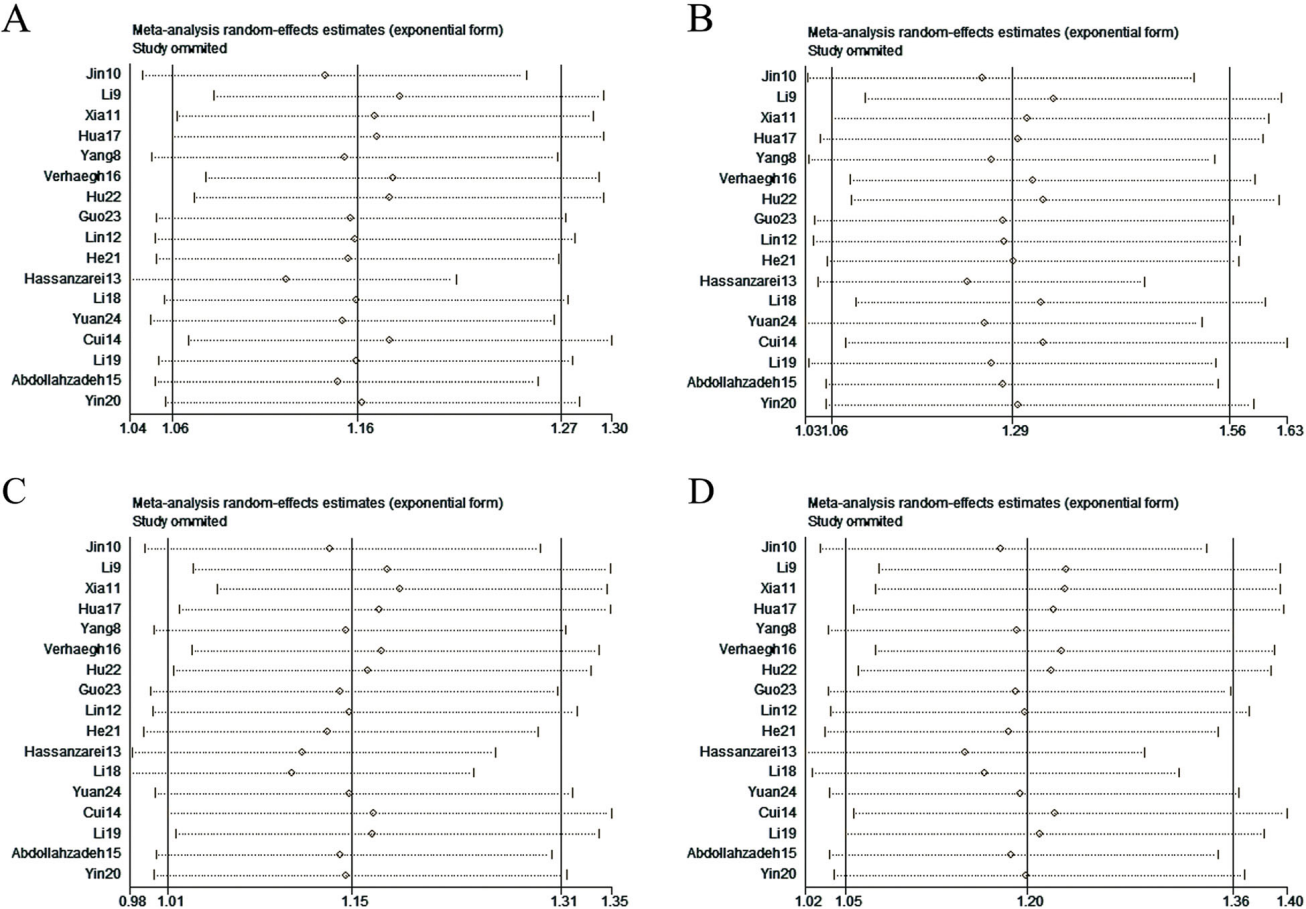
Sensitivity analysis through deletion of one study at a time to reflect the influence of the individual dataset to the pooled ORs in H19 rs217727 polymorphism. **a** Allelic model (T vs. C), **b** homozygote model (TT vs. CC). **c** heterozygote model (CT vs. CC). **d** Dominant model [(CT + TT) vs. CC]

**Fig. 5:**
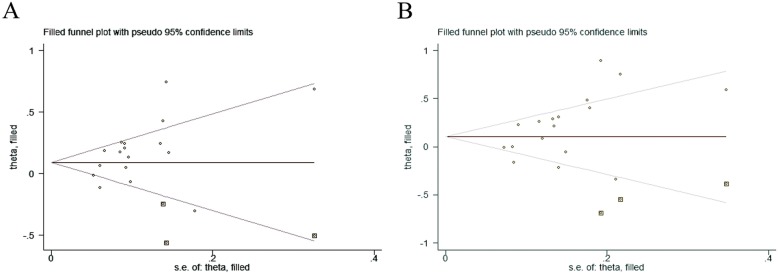
Trim and fill method’s funnel plot of the association between H19 rs217727 polymorphism and cancer risk. **a** Allelic model (T vs. C), **b** dominant model [(CT + TT) vs. CC]

Sensitivity analysis was performed by omitting each and every included studies. As shown in Fig. 4, the results indicated that the pooled ORs were not subjective to change, which indicated the stability of our study. To assess the publication bias for the studies, both the Egger’s test and Begg’s funnel plot were performed. Publication bias was found in allelic model (*P* = 0.04), heterozygote model (*P* = 0.05), dominant model (*P* = 0.03). Trim and fill method was used to identify and correct the publication bias. Before and after the trim, ORs does not change, which indicates that despite the publication bias in this study, the publication bias has little impact, and the research results are robust and reliable. The trim and fill method’s funnel plot is shown in Fig. 5.

## Discussion

In recent years, many studies have focused on the relationship between genotype and phenotype, and the personalized prevention and treatment of cancer based on genetic information is the current research trend and hotspot [28]. SNP is the most common type of gene polymorphism, which may affect gene expression and function through indirect influence of related transcription factors or microRNAs, and further participate in the occurrence and development of tumors. LncRNA *H19* has been widely recognized for its aberrant expression profile and role in carcinogenesis, and it is suggested to be a novel biomarker for the diagnosis of cancer [29, 30]. In addition, numerous studies have focused on the relation between *H19* SNPs and cancer susceptibility. A study conducted by Yang et al. revealed that the TT + CT genotype of rs2839698 could increase the risk of hepatocellular cancer [31]. In terms of *H19* rs217727, it was found to increase the risk of breast cancer [12, 13, 15]. Further functional experiments found that the expression level of *H19* in breast cancer tissues was higher than that in normal tissues, and rs217727 CT or TT genotype was helpful to improve the expression level of *H19* (P<0.001, 12]. However, no significant correlation was found in the study conducted by Xia et al. [11]. Furthermore, a study [17] included 1049 cancer cases and 1399 controls, showed that the AA genotype increased the risk of bladder cancer up to 1.31 times compared with the GG/GA genotype. Similarly, a positive relation was also found in gastric cancer [8] and cervical cancer [10]. However, in another study it was demonstrated that rs217727 did not associate with risk of colorectal cancer in additive model [9]. The results were inconsistent and inconclusive, and might be due to the limited sample size, the difference in genetic background, or the type of cancer. Therefore, in this study, we performed meta-analysis to comprehensively evaluate the association between *H19* SNPs and susceptibility to cancer.

In the current meta-analysis, which included 17 case-control studies, people with the T, TT, CT and CT + TT genotypes of SNP rs217727 got a higher risk of cancer. Similarly, subgroup analysis based on ethnicity, type of cancer and genotyping method showed an increased risk for all genetic models in Asian, oral squamous cell carcinoma and genotyping approach according to MassArray. In addition, the risk of lung cancer increased in the allelic, homozygote models, and for breast cancer, the risk increased in the allelic model. The significant association was also found in allelic, homozygote, heterozygote and dominant models in the subgroup of hospital-based controls, as well as in allelic, homozygote, dominant and recessive models in the subgroup with a sample size of more than 500. Overall, the study revealed that *H19* rs217727 might increase the risk of cancer. Interestingly, we also found that smoking was not significantly associated with the development of cancer in *H19* rs217727.

Our results differ from those previously published [32–35]. Lv et al. [32] and Li et al. [35] included 5 studies and concluded that the rs217727 C > T might not be associated with the risk of cancer. Chu et al [33] used differently 3 genetic models, and the pooled results showed that the heterozygote and dominant model of rs217727 appeared to be a protective factor to cancer in hospital-based controls, as well as in the subgroup of population-based controls. Lu’s study, which included 4 literatures, subgroup analyses only stratified by genotyping approach and failed to reveal the relationship between rs217727 C > T and cancer risk [34]. The increased sample size and newly incorporated studies in our study may explain this difference. For the relation observed in subgroup meta-analysis, but not in overall meta-analysis, there are several possibilities to explain this difference, such as differences in genetic background, and the complex process of cancer formation. Interestingly, we also found that *H19* rs217727 was associated with a neoplastic predisposition, and had little to do with smoking.

Our meta-analysis has several limitations, which should be addressed. First, despite the comprehensive analysis that has been performed to determine a possible relation, potential covariates (age, sex, drinking status, and smoking status) cannot be extracted from all included cases. Thus, the pooled results were based on unadjusted data. Second, the sample size of this study is still limited, which may reduce the power of analysis. Therefore, the data should be validated in a larger study. Third, only English databases were used in our search, which may affect our results. If literatures of other languages were included in this study, it would be possible that additional estimations could have been conducted. Finally, after subgroup analyses, heterogeneity could still be observed in a variety of SNPs, therefore, our conclusions should be treated with caution.

## Conclusions

LncRNA *H19* rs217727 could increase cancer risk in overall population, as well as in Asians, subgroups for genotyping based on MassArray, oral squamous cell carcinoma, lung cancer, breast cancer, hospital-based controls and subgroups with a case sample size ≥500. Because of the limitations in our study, well-designed studies with a larger sample size, and adjusted risk factors are required to further confirm the conclusions.

## Supplementary information

**Additional file 1.** PubMed search strategy.

## Data Availability

The datasets generated and analyzed during the present study are available from the corresponding author upon reasonable request.

## Abbreviations

CI: Confidence interval
EBV: Epstein-Barr virus
GWAS: Genome-wide association studies
HOTAIR: HOX transcript antisense RNA
HPV: Human papillomavirus
HWE: Hardy-Weinberg Equilibrium
IGF2: Insulin-like growth factor 2
LncRNA: Long non-coding RNA
NOS: Newcastle Ottawa Scale
OR: Odds ratio
PRISMA: Preferred Reporting Items for Systematic Reviews and Meta-Analyses
SNP: Single nucleotide polymorphism
