# Supplementary material for: Association between lncRNA H19 rs217727 polymorphism and the risk of cancer: an updated meta-analysis
Source: BMC Med Genet. 2019 Nov 21;20:186. doi: 10.1186/s12881-019-0904-x (PMC6873771; doi:10.1186/s12881-019-0904-x)
Supplement: Supplementary file 1 — Additional file 1. PubMed search strategy. [file 12881_2019_904_MOESM1_ESM.docx]

**Additonal file 1: PubMed search strategy**

Search ((((((((((("Polymorphism, Genetic"[Mesh]) OR Genetic Polymorphisms[Title/Abstract]) OR Genetic Polymorphism[Title/Abstract]) OR Polymorphisms (Genetics)[Title/Abstract]) OR "Mutation"[Mesh]) OR Mutations[Title/Abstract]) OR variation[Title/Abstract]) OR variant[Title/Abstract]) OR Polymorphism[Title/Abstract])) AND ((((((long Noncoding RNA H19[Title/Abstract]) OR lncRNA H19[Title/Abstract]) OR long non-coding RNA H19[Title/Abstract]) OR H19[Title/Abstract]) OR "H19 long non-coding RNA" [Supplementary Concept]) OR H19 RNA[Title/Abstract])) AND ((((((((((((((("Neoplasms"[Mesh]) OR Neoplasia[Title/Abstract]) OR Neoplasias[Title/Abstract]) OR Neoplasm[Title/Abstract]) OR Tumors[Title/Abstract]) OR Tumor[Title/Abstract]) OR Cancer[Title/Abstract]) OR Cancers[Title/Abstract]) OR Malignant Neoplasms[Title/Abstract]) OR Malignant Neoplasm[Title/Abstract]) OR “Neoplasms, Malignant”[Title/Abstract]) OR Malignancy[Title/Abstract]) OR Malignancies[Title/Abstract]) OR Benign Neoplasms[Title/Abstract]) OR Benign Neoplasm[Title/Abstract])
